# Aqueous Extract of Tomato (*Solanum lycopersicum* L.) and Ferulic Acid Reduce the Expression of TNF-α and IL-1β in LPS-Activated Macrophages

**DOI:** 10.3390/molecules200815319

**Published:** 2015-08-21

**Authors:** Simón Navarrete, Marcelo Alarcón, Iván Palomo

**Affiliations:** 1 Immunology and Haematology Laboratory, Faculty of Health Sciences, Universidad de Talca, Talca 3460000, Chile; 2 Department of Clinical Biochemistry and Immunohematology, Faculty of Health Sciences, Interdisciplinary Excellence Research Program on Healthy Aging (PIEI-ES), Universidad de Talca, Talca 3460000, Chile; E-Mail: malarcon@utalca.cl; 3 Centro de Estudios en Alimentos Procesados (CEAP), CONICYT-Regional, Gore Maule R09I2001, Chile

**Keywords:** inflammation, macrophages, proinflammatory cytokines, NF-κB

## Abstract

Acute inflammation is essential for defending the body against pathogens; however, when inflammation becomes chronic, it is harmful to the body and is part of the pathophysiology of various diseases such as Diabetes Mellitus type 2 (DM2) and Cardiovascular Disease (CVD) among others. In chronic inflammation macrophages play an important role, mainly through the secretion of proinflammatory cytokines such as Tumor necrosis factor (TNF)-α and Interleukin (IL)-1β, explained in part by activation of the Toll-like receptor 4 (TLR4), a signaling pathway which culminates in the activation of Nuclear factor (NF)-κB, an important transcription factor in the expression of these proinflammatory genes. On the other hand, the benefits on health of a diet rich in fruit and vegetables are well described. In this work, the effects of aqueous extract of tomato and ferulic acid on the expression of proinflammatory cytokines in LPS activated monocyte-derived THP-1 macrophages were investigated. In addition, using Western blot, we investigated whether the inhibition was due to the interference on activation of NF-κB. We found that both the tomato extract and ferulic acid presented inhibitory activity on the expression of TNF-α and IL-1β cytokine by inhibiting the activation of NF-κB. The current results suggest that tomatoes and ferulic acid may contribute to prevention of chronic inflammatory diseases.

## 1. Introduction

Acute inflammation is the first response of the organism to control an insulting agent [1]. Macrophages are major players at the beginning of this process because they recognize microorganisms and dead cells, and produce various proinflammatory mediators such as Interleukin (IL)-1, TNF-α, Interleukin (IL)-6, Prostaglandin E2 (PGE2) and NO [2]. These mediators activate endothelial cells and allow the arrival of more cells such as monocytes and neutrophils that fight the pathogen and release more inflammatory mediators [3,4,5].

THP-1 cells, a human monocytic cell line [6], can differentiate into macrophages by treatment with phorbol esters such as phorbol 12-myristate 13 acetate (PMA), that activates protein kinase C (PKC), mimicking the physiological activator diacylglycerol (DAG) [7]. Macrophages have different pathogen recognition receptors, including the main group of Toll-like receptor (TLR) [8]. Lipopolysaccharide (LPS) is recognized by TLR4 and activates a signaling pathway independent or Myeloid differentiation primary response gene 88 (MyD88)-dependent leading to the activation of NF-κB and proinflammatory cytokine production. This signaling pathway is finely regulated to avoid damage to the organism induced by inflammation [9]. On the other hand, chronic inflammation is a maladapatative process, involved in the pathophysiology of obesity, insulin resistance, DM2 and CVD [10,11].

Functional foods are those found in a natural or processed form, that contain components that exert beneficial health effects that go beyond nutrition [12]. These functional foods possess bioactive compounds such as flavonoids, hydroxycinnamic acids, tannins, *etc.* [13]. There is epidemiological evidence that demonstrates the protective role of diets rich in fruit, vegetables, legumes, whole grains, nuts, unsaturated fats and fish on cancer and CVD [14,15]. It has been reported that tomato has beneficial effects on health, such as antiplatelet and antioxidant effects [16,17], besides cancer and CVD prevention [18]. Additionally, it has been shown to reduce systemic inflammation in women with obesity or overweight by decreasing serum proinflammatory cytokines [19]. In addition, tomato extract supplementation has been reported to decrease hepatic inflammation and total cholesterol in mice treated with a high-fat diet [20]. Ferulic acid, a hydroxycinnamic acid derivative, is present in tomatoes [21]; its concentration in aqueous tomato extract is approximately 4786.7 ± 3 mg/kg [22]. This molecule has been found to attenuate cerebral ischemia in mice [23]. It has also been reported that it attenuates the expression of iNOS in LPS-stimulated RAW 264.7 macrophages [24] and CXCL2 in the same cell line in response to respiratory syncytial virus infection [25]. In addition, it has been described that ferulic acid has protective effect on the endothelium, attenuating the expression of adhesion molecules in gamma-radiated human umbilical vascular endothelial cells [26]. In this work, we evaluated the effect of tomato extract and ferulic acid on the expression of proinflammatory cytokines TNF-α and IL-1β and its impact on the activation of NF-κB in LPS-activated macrophages.

## 2. Results

### 2.1. Viability Study in Macrophages Incubated with Aqueous Tomato Extract and Ferulic Acid

Table 1 shows the viability of macrophages incubated with tomato extract and ferulic acid, inactivated and activated with LPS. No significant differences between viability of cells incubated with tomato extract or ferulic acid, activated and inactivated, and the macrophages without aqueous tomato extract neither ferulic acid.

**Table 1:** Aqueous tomato extract and ferulic acid does not affect the viability of macrophages.

Aqueous Tomato Extract (mg/mL)	Viability by Trypan Blue (%)		Viability by MTT (%)	
	Unactivated	Activated	Unactivated	Activated
0	99.3 ± 0.8	96.9 ± 2.4	100.0	98.0 ± 1.3
0.1	94.0 ± 0.5	98.4 ± 1.0	96.9 ± 3.4	96.9 ± 2.6
0.5	98.2 ± 0.6	95.4 ± 4.0	99.1 ± 0.4	96.5 ± 2.1
1.0	97.7 ± 1.1	96.9 ± 2.4	95.4 ± 4.2	95.4 ± 4.0
**Ferulic Acid (µM)**				
0	99.5 ± 0.5	96.9 ± 2.4	100.0	98.0 ± 1.3
125	97.9 ± 0,7	97.0 ± 1.1	96.0 ± 0.4	94.6 ± 0.6
250	94.9 ± 1.0	95.0 ± 0.6	95.1 ± 0.3	98.0 ± 0.7
500	96.3 ± 1.0	96.9 ± 2.4	97.7 ± 2.1	95.4 ± 4.0

Means ± S.E.M.; MTT, 3-(4,5-dimethylthiazol-2-yl)-2,5-diphenyltetrazolium bromide.

### 2.2. Gene Expression Kinetics of TNF-α and IL-1β in LPS-Activated Macrophages and Effect of Aqueous Tomato Extract and Ferulic Acid on the Expression of These Cytokines

**Figure 1:**
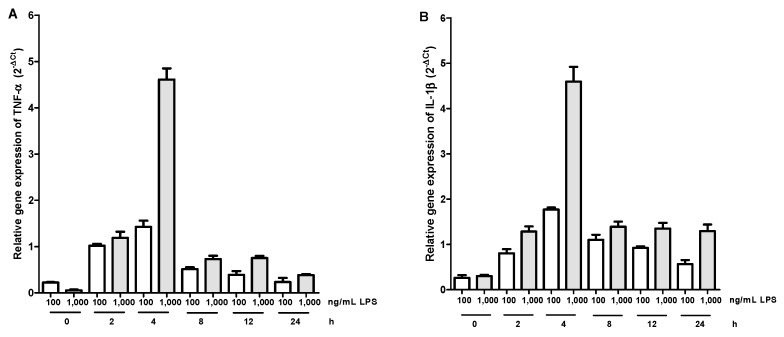
Gene expression kinetics of TNF-α (**A**) and IL-1β (**B**). It is observed that the peak of expression for both cytokines was 4 h.

**Figure 2:**
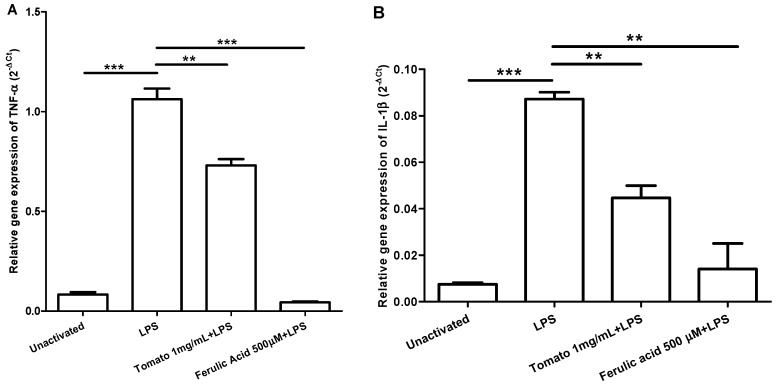
Aqueous tomato extract and ferulic acid inhibit expression of TNF-α (**A**) and IL-1β (**B**) in LPS-activated macrophages. LPS, lipopolysaccharide. ** significant at the *p* < 0.01 level, *** significant at the *p* < 0.001 level.

The peak for TNF-α expression and IL-1β occurred at four hours post LPS activation at the two concentrations evaluated, however, the highest expression was obtained with LPS 1000 ng/mL (Figure 1). Figure 2 shows the relative expression of TNF-α and IL-1β in unactivated macrophages, activated with LPS, and incubated with aqueous tomato extract and ferulic acid for one hour and then activated with LPS. Macrophages incubated with aqueous tomato extract and ferulic acid had a lower expression of both cytokines compared to control (activated with LPS) (*p* <0.05).

### 2.3. Kinetics of NF-κB Activation in LPS-Activated Macrophages and Effect of Aqueous Tomato Extract and Ferulic Acid on Activation of This Transcription Factor

**Figure 3:**
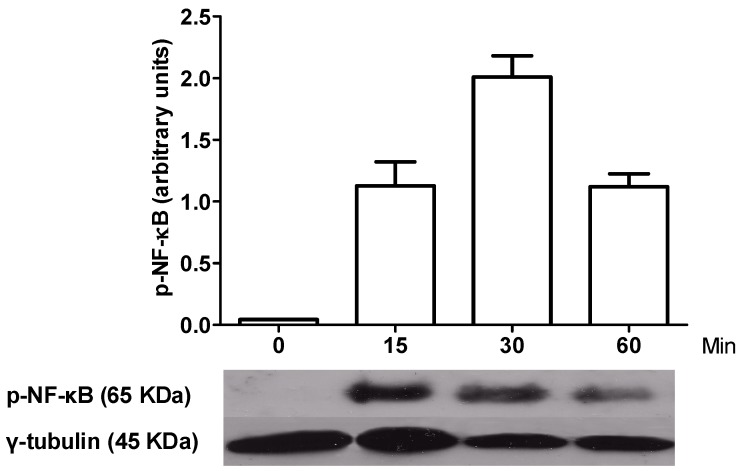
Kinetics of activation of NF-κB in LPS-activated macrophages. It is observed that the peak of NF-κB activation occurred at 30 min after the challenge with LPS.

**Figure 4:**
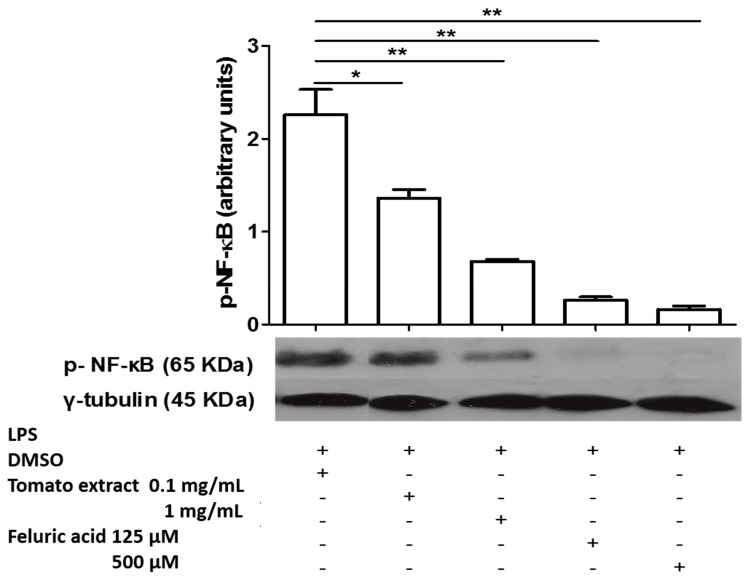
Extract tomato and Ferulic Acid inhibit the activation of NF-κB in LPS-activated macrophages. LPS, lipopolysaccharide; DMSO, dimethylsulfoxide, * significant at the *p* < 0.05 level, ** significant at the *p* < 0.01 level.

Figure 3 shows that the peak activation of NF-κB was at 30 min post LPS in macrophages. Figure 4 shows that both tomato extract and ferulic acid inhibit NF-κB phosphorylation in LPS activated (*p* < 0.05) macrophages, previously incubated 1 h with these components, compared to control (activated macrophages).

## 3. Discussion

The acute inflammatory response is essential to fight infections, but when this response becomes chronic, it is harmful to the organism as part of pathophysiology of diseases such as type 2 diabetes and CVD [27]. In this response, macrophages play an important role, through the production of proinflammatory cytokines. It has been reported that in obese people there is an increase in macrophage infiltration in the adipose tissue [28] and these macrophages polarize to a proinflammatory phenotype (M1) [29]. The increase in TNF-α promotes additional proinflammatory cytokine secretion and reduces anti-inflammatory cytokines such as adiponectin. TNF-α promotes insulin resistance by inhibiting the IRS1 (insulin receptor substrate 1) signaling pathway [30,31]. Additionally, IL-1β is toxic for the β-pancreatic cells causing apoptosis, thereby decreasing the production of insulin [32]. It is known that a diet high in fruits, vegetables, legumes, cereals, and fish, among others is beneficial to health [33,34]. In this study, we found that the aqueous extract of tomato and ferulic acid inhibit the expression of TNF-α and IL-1β in LPS-stimulated macrophages, through the inhibition of NF-κB.

In the literature, there are no reports regarding the effect of tomato extract on the proinflammatory cytokine production in macrophages derived from the cell line THP-1, activated with LPS. Nevertheless, there are studies reporting that some components of tomato inhibit inflammatory mediators. Rafi *et al.* reported that lycopene inhibits NO production in murine macrophage cell line RAW 264.7, through the inhibition of the expression of iNOS gene, expression that is coordinated primarily by NF-κB [35]. Meanwhile, Dou *et al.* showed that the flavonoid naringenin, found in tomatoes, inhibits the production of several proinflammatory mediators such as iNOS, ICAM-1, Cox2, TNF-α and IL-6 in a murine model of intestinal inflammation, through attenuation of NF-κB activation [36]. These findings are consistent with our observation that tomato extract contains several components able to inhibit cytokine synthesis.

In the same vein, we verified that ferulic acid, a derivative of hydroxycinnamic acid present in tomatoes, had the same effect as tomato extract. In this regard, Sakai *et al.* reported that ferulic acid and isoferulic acid exerted an inhibitory effect on the production of CXCL2 in RAW 264.7 macrophages [25], where this chemokine may have been induced by stimulation with LPS [37]. Furthermore, Kim *et al.* showed that ferulic acid inhibits the expression of iNOS in RAW 264.7 macrophages stimulated with LPS again, through the inhibition of NF-κB [24]. These studies support our results, since they describe inhibition of the expression of effectors produced by the activation of the same inflammatory pathway. Furthermore, it has also been reported that a ferulic acid derivative, ferulic acid ethyl ester, inhibit the translocation of NF-κB in RAW 264.7 macrophages stimulated with LPS [38]. Nagasaka *et al.*, found that cycloartenil ferulate inhibits expression of iNOS and COX-2 by inhibition of NF-κB [39]. Other studies have also shown that some ferulic acid derivatives have potent inhibitory effects on inflammation, while ferulic acid showed less potency. Murakami *et al.* found that FA15, a ferulic acid derivative markedly inhibited the expression of iNOS and COX-2 and TNF-α secretion by inhibition of IkB degradation in RAW 264.7 macrophages, while ferulic acid poorly exhibited this effect [40]. Roncheti *et al.* also showed that NCX 2057, another ferulic acid derivative, inhibited the expression of iNOS in RAW 264.7 macrophages activated with LPS/IFN-γ, in contrast to ferulic acid [41]. Finally, Hirata *et al.* showed that ferulic acid derivatives have inhibitory effect on the expression of COX-2 in RAW 264.7 macrophages stimulated with LPS, but ferulic acid has no such effect. [42].

Importantly, LPS-signaling that is activated in macrophages via TLR4 is complex and involves several kinases. After binding to its receptor, the signal is transmitted by different adapter proteins: TIRAP (Toll-interleukin 1 receptor domain-containing adapter protein) and MyD88 (Myeloid differentiation primary response gene 88) driving the MyD88-dependent pathway, while TRAM (TRIF-related adaptor molecule) and TRIF (TIR-domain-containing adapter-inducing interferon-β) driving the MyD88-independent pathway [43]. The first pathway follows the activation of IRAK 1 and 4 (IL-1R activated kinases 1 and 4), TRAF6 (Tumor necrosis factor (TNF)-receptor-associated factor 6) and TAK1 (Transforming growth factor-β-activated kinase 1). The MyD88-independent pathway activation continues with RIP-1 (Receptor interacting protein 1) and TRAF3. Finally, the MyD88-dependent pathway converges in IKK (IκB kinase) and MAPK (Mitogen-activated protein kinases) activation and subsequent activation of NF-κB and activator protein (AP)-1 [9]. Given the complexity of the signaling pathway that activates NF-κB additional studies are needed to establish the stage where the tomato extract and ferulic acid produce inhibition.

## 4. Experimental Section

### 4.1. Cell Culture and Macrophage Differentiation

The THP-1 cells were expanded in culture medium RPMI 1640 1X (Gibco, Grand Island, NY, USA) supplemented with inactivated fetal bovine serum (FBS; Cellgro, Manassas, VA, USA) to 10%, penicillin/streptomycin (Gibco) to 1% and 2-Mercaptoethanol (Amresco, Solon, OH, USA) 0.05 mM, in a humidified incubator at 37 °C and 5% CO_2_. The culture medium was renewed every 3 days and the assays were performed at passage 10.

Differentiation of THP-1 cells to macrophage was induced with phorbol 12-myristate 13 acetate (PMA, Sigma-Aldrich, St. Louis, MO, USA) at final concentration of 160 ng/mL, in 24 well plates, where cell concentration was 1 × 10^6^ cells/mL and the final volume was 500 µL. Cells were incubated 48 h, the culture medium was renewed, and incubated for an additional 24 h. After this period of time, macrophages M0 were obtained, according to the literature [25].

### 4.2. Aqueous Extract of Tomato and Ferulic Acid

The aqueous extract of tomato was prepared as follows: small pieces of red tomato pulp (free of seeds) were comminuted in a blender, and the resulting mash was filtered through gauze twice. The liquid obtained was lyophilized and stored at −80 °C. At the time of use, this was diluted in distilled water, sonicated and filtered (0.22 µm pore size). The final concentrations used were 0.1, 0.5 and 1 mg/mL. Commercial ferulic acid (Sigma-Aldrich) was used and prepared in distilled water plus DMSO (concentration in culture <1%), sonicated and then filtered through a syringe filter 0.22 µm. Final concentrations used were 125, 250 and 500 µM.

### 4.3. Viability Study by Trypan Blue Exclusion and Reduction of Bromide 3-(4,5-Dimethyl-2-thiazoyl)-2,5-difeniltetrazólico (MTT)

Macrophages were incubated with tomato extracts and ferulic acid to the maximum concentration used (1 mg/mL and 500 µM, respectively) or DMSO for 24 h. To determine viability by Trypan Blue exclusion, 50 µL of 1X Trypsin (SAFC Biosciences, Inc., Lenexa, KS, USA) was added to each well, incubated at 37 °C for 3 min and resuspended in 500 µL of medium. An aliquot of 100 µL of cell suspension was mixed with 800 µL of sterile PBS and 100 µL of 4% Trypan Blue (Gibco). Then the mixture was homogenized and loaded in a Neubauer chamber. Viability was calculated as follows: % viable cells = [1.00 − (number of blue cells/total cells)] × 100. To determine viability by MTT to each well were added 200 µL of MTT solution (50 µL of MTT 5 mg/mL and 150 µL of RPMI supplemented), then incubated for 4 h at 37 °C, washed with sterile PBS and 200 µL of lysate solution 0.04 N is added (10 µL of HCl to 3000 µL of isopropanol) and the lysate was centrifuged at 12,000 rpm for 5 min and absorbance was measured between 570 and 630 nm. Control absorbance, macrophages without aqueous extract tomato or ferulic acid, is considered 100% viability.

### 4.4. Expression of Proinflammatory Cytokines

The peak of expression for TNF-α and IL-1β was determined in addition to the concentration of LPS used. First macrophages were activated with LPS (*E. coli* 0111: B4, Sigma-Aldrich) at 100 ng/mL or 1000 ng/mL and incubated for 0, 2, 4, 8, 12 and 24 h in culture oven. mRNA extraction was done with the EZNA^®^ kit (Omega Bio-Tek, Norcross, GA, USA), according to manufacturer’s instructions. To synthesize cDNA, the AffinityScript QPCR cDNA Synthesis kit was used. The real-time PCR was done with the Brilliant SYBR Green QPCR Master Mix. The primers, forward and reverse, respectively, were as follows: TNF-α (CCGTCTCCTACCAGACCAAGG, CTGGAAGACCCCTCCCAGATAG), IL-1β (CCCACAGACCTTCCAGGAGA, CGGAGCGTGCAGTTCAGTG) and for the reference gene β2-microglobulin (GCTCCGTGGCCTTAGCTGT, ACGTGAGTAAACCTGAATCTTTGGA), all used to the concentration of 400 nM and 40 ng of cDNA were used in a final volume of 25 μL. An initial cycle of 10 min at 95 °C was programmed followed by 40 cycles of 30 s at 95 °C, 1 min at 60 °C and 30 s at 72 °C. The relative gene expression for both cytokines was calculated by 2^−∆Ct^ method (2^−^^(Ct(gene^ °^f interest) − Ct(reference gene))^). To evaluate the effect of tomato extract and ferulic acid on the expression of TNF-α and IL-1β, macrophages were incubated with different concentrations of these compounds or DMSO at final concentration of 1% for 1 h. Then, macrophages were activated with LPS 1000 ng/mL and incubated for 4 h to proceed as mentioned above.

### 4.5. Western Blotting for Phospho-NF-κB p65 (Ser536)

First macrophages were activated at 0, 15, 30 and 60 min with LPS a 1000 ng/mL, to determine the peak of activation for NF-κB. Then, the cells were lysed with RIPA buffer (Sigma-Aldrich). Protein concentration was measured by BCA method (BCA Protein Assay TM Pierce, Thermo Scientific, Rockford, IL, USA) and 2 µg of total protein was loaded on SDS-PAGE. The primary antibody rabbit IgG anti-Phospho-NF-κB p65 (Ser536) (Cell Signaling Technology, Beverly, MA, USA) and the loading control antibody rabbit IgG anti-γ-Tubulin (Thermo Scientific) were used. As secondary antibody, anti-rabbit IgG peroxidase conjugate (Sigma, Saint Louis, MO, USA) was used. After incubation with the secondary antibody, the membrane was incubated with ECL Western blotting Substrate Pierce (Thermo Scientific) for one min. Then, the film (CL-XPosure™ Film, Thermo Scientific) was exposed to the membrane for 15 min and developed. To evaluate the effect of aqueous tomato extract and ferulic acid on activation of NF-κB, macrophages were incubated with different concentrations of these or DMSO at final concentration of 1% for 1 h, and then activated with LPS to 1000 ng/mL for 30 min.

### 4.6. Statistical Analysis

Data were analyzed using GraphPad Prism 5 software for Windows. Normality test was made and the results are expressed as mean plus standard error. To analyze the statistical significance of differences between control and test values obtained *t*-Student was used considered statistically significant differences with *p* < 0.05 (* *p* < 0.05, ** *p* < 0.01 and *** *p* < 0.001).

## 5. Conclusions

The aqueous extract of tomato has an inhibitory effect on the production of TNF-α and IL-1β in LPS-activated macrophages. This effect is due to the action of several compounds present in tomatoes having an anti-inflammatory effect. One of these compounds is ferulic acid, which also inhibits the expression of these cytokines. This inhibition is due to inhibition of NF-κB activation. However, the signaling pathway that leads to the activation of this transcription factor comprises several protein kinases. Therefore, further studies are needed to identify the exact target. Since these proinflammatory cytokines exert different functions in the pathology of chronic diseases, consumption of these elements may be beneficial in preventing the onset of diseases such as diabetes and CVD or mitigate their effects.
